# Morphological and Genetic Assessments of Coyote Diet in Qualla Boundary, North Carolina, Show Interaction with Humans

**DOI:** 10.3390/ani15050741

**Published:** 2025-03-05

**Authors:** Caitlin Miller, Donald Linzey, Eric Hallerman

**Affiliations:** Department of Fish and Wildlife Conservation, Virginia Polytechnic Institute and State University, Blacksburg, VA 24061, USA; cait1inm@vt.edu (C.M.); dlinzey@vt.edu (D.L.)

**Keywords:** DNA barcoding, diet study, coyotes, Southeastern United States

## Abstract

Throughout the 20th century, coyotes (*Canis latrans*) expanded from their historical geographic range west of the Mississippi River to a current range of almost all of North America. Over the course of this expansion, coyotes have demonstrated diverse and variable omnivorous diets that change with the food resources available. This study examined the stomach contents of 25 coyotes in an area where they are relatively new, the Qualla Boundary in North Carolina, to better understand the diets of coyotes in this area. A combination of morphological identification and DNA barcoding was used to characterize the stomach contents of coyotes. Both plant and animal material were identified from anthropogenic and natural sources, the latter including native mammals. This study provides one example of the breadth and flexibility of coyote diets and helps build an understanding of how coyotes can adapt to new conditions.

## Simple Summary

Coyotes (*Canis latrans*) have expanded their range to the Southeastern United States, where they have adapted to exploit both natural and anthropogenic food resources. Their expansion into the Qualla Boundary, a land trust for the Eastern Band of Cherokee Indians, may have negatively impacted native mammals and has led to human–wildlife conflict, leading to the lethal management of coyotes. We examined the stomach contents of 25 culled coyotes using visual examination and DNA barcoding to identify food items. We observed a wide range of plant and animal items, showing that coyotes exploited both wild and anthropogenic food resources.

## 1. Introduction

Over the course of the 20th century, coyotes (*Canis latrans*) expanded from their historical geographical range west of the Mississippi River to a current range of almost all of North America [1,2,3,4]. The eastward spread of coyotes has been explained by both natural expansions and introductions by humans. Natural expansions have been explained by the decline of native eastern canid species, such as the eastern gray wolf (*Canis lupus lycaon*) and red wolf (*Canis rufus*), which removed competition and created an open ecological niche for coyotes [2,3]. Coyotes have also been introduced to the Eastern United States by people hoping to make them pets, or by fox hunters who were mistakenly sent coyotes instead of fox pups. These transported coyotes have then either escaped or been purposefully released [5,6,7]. Particularly in the southern states, many coyotes were liberated by fox hunters after having had similar-appearing coyote pups shipped to them instead of fox pups [5,6,7]. The U.S. Fish and Wildlife Service has documented 20 different points in the Southeastern United States where coyotes were released by people who planned to hunt them with hounds; three of the release sites were in Tennessee, including Sequatchie County in the southeast [5,6]. Coyotes first appeared in North Carolina at some point in the 1930s–1940s [8], with the first positive identification of a killed coyote on the Qualla Boundary in 1947 [9]. Eastward movement has presented new challenges for coyotes, such as hunting in forests with greater undergrowth [10]. The clearing of forested land created ecotones, where fields abut fencerows, thereby increasing habitat availability for their primary food items such as rodents and rabbits [5,6]. Despite ecosystem differences, coyotes have become an established species in the Southeastern United States [3]. One factor that has facilitated coyotes’ movement into both new geographic areas and more urban areas is a remarkable degree of dietary plasticity. Coyotes have a meat-based but omnivorous diet that varies across season, individual coyote, and geographic area [11,12,13,14,15,16]. Previous diet studies of coyotes in the Southeastern United States found that their diets are primarily based on white-tailed deer (*Odocoileus virginianus*), rodents, lagomorphs, and fruits, with eastern coyotes eating more ungulates and vegetation than western coyotes [12,16,17]. In a meta-analysis of coyote diets across North America, greater dietary diversity was observed in spring and winter and in areas with more significant human presence [16]. Coyotes have regularly been observed consuming previously undocumented food sources such as commercial sunflower (*Helianthus* spp.) seed and pinyon pine (*Pinus edulis*) nuts [13,18]. The ability to adapt to new food items can apply to both natural sources [18] and anthropogenic food sources, including the consumption of domestic cats (*Felis catus*) [11]. The flexibility and diversity of coyote diets mean that studies across different areas, seasons, and individual coyotes can all provide unique insights into coyote feeding patterns.

Coyotes have expanded their range to the Southeastern United States, where they have adapted to exploit both natural and anthropogenic food resources. Their expansion into the Qualla Boundary, a land trust for the Eastern Band of Cherokee Indians, may have negatively impacted native mammals and led to human–wildlife conflict, leading in turn to the lethal management of coyotes. Sixty-eight species of mammals are found in Great Smoky Mountains National Park [6], which is adjacent to the Qualla Boundary, including many species that are likely prey or competitors of coyotes. Within the National Park, the primary diet items of coyotes of animal origin are thought to be rabbits (*Sylvilagus* sp.), rodents, and carrion, as well as newborn fawns, possibly elk (*Cervus canadensis*) calves [5], and wild piglets (*Sus scrofa*) [6]. Wherever coyotes establish themselves in large numbers, red foxes *Vulpes vulpes* disappear, in contrast to gray foxes *Urocyon cinereoargenteus*, which may coexist more easily with coyotes [19]; coyotes may suppress fox population growth by aggression and competition for food [20]. Kumar et al. [21] and coauthor Linzey have noted declining small mammal populations in portions of Great Smoky Mountains National Park since the 1980s. In the context of this study system, coyote–human conflict issues include impacts of coyotes on the native fauna, which is highly valued by the Native American community, and the apparent consumption of garbage by coyotes, creating a nuisance and fear of impacts upon domestic animals. These conflicts have led to a program of the lethal culling of coyotes by wildlife managers. The Cherokees began reintroducing white-tailed deer onto the Qualla Boundary in 2014, and the wildlife manager was interested in whether or not coyotes would prey on the newly introduced deer and their fawns. Against this background, we characterized the diets of coyotes on the Qualla Boundary in North Carolina by examining stomach contents using both morphological identification and DNA barcoding.

The morphological identification of stomach contents has long been practiced, but even when performed by an experienced individual, many items remain unidentifiable. DNA barcoding is a molecular genetic protocol that can support the species-level identification of samples using diagnostic DNA sequences. The DNA barcoding of vertebrates utilizes the mitochondrial cytochrome c oxidase I (*COI*) gene because it does not have introns, has limited exposure to recombination, is present in most phyla, and has a rapid rate of evolutionary change among species but relatively low variation within species [22]. DNA barcoding can be used to help identify partially digested dietary items, with the inclusion of DNA barcoding identification increasing the proportion of items that could be identified by 66.1% compared to morphological identification alone in one study of diet habits [23].

The combined use of morphological and genetic identification should help identify a high percentage of coyote stomach contents obtained in dissections. Using this approach, we aimed to assess whether predation by coyotes is a driver of the decline of local mammals in the Qualla Boundary. We hypothesized that if coyotes were responsible for this decline, we should find the frequent presence of native mammals in their stomach contents. Many coyotes were culled near human settlements, suggesting that the animals were feeding upon human refuse; if this were the case, we hypothesized that we would find human food waste items in their stomach contents. These results would also provide information about how coyotes’ flexible diets may be changing to deal with the specific resources present in our study site and could contribute to a general understanding of how broad coyote diets can become. These results would provide information about coyote behavior in the Qualla Boundary and adjacent Great Smoky Mountains through a greater understanding of their diet composition. This information will contribute to our understanding of coyotes’ versatility and adaptability and help provide insights into how they might adapt to other new areas.

## 2. Materials and Methods

### 2.1. Study Area

**Figure 1:**
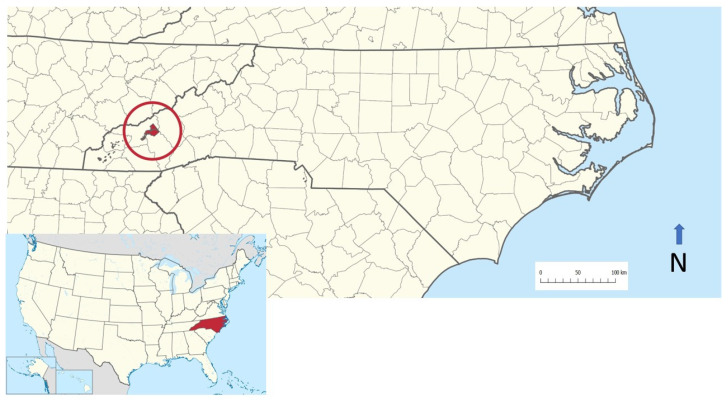
Location of Qualla Boundary within North Carolina, Southeastern USA (A.W. McPhee, https://en.wikipedia.org/wiki/Qualla_Boundary, accessed on 18 October 2024). The distance bar shows 100 km.

This study was conducted on the Qualla Boundary (Figure 1), located at 35°30′ N 83°16′ W, which is a territory held as a land trust by the United States government for the federally recognized Eastern Band of Cherokee Indians, who reside in western North Carolina. The main part of the Qualla Boundary lies in the eastern Swain and northern Jackson counties just south of Great Smoky Mountains National Park, and a small portion extends eastward into Haywood County. In total, the Qualla Boundary is 213.9 km^2^ in area, with 8816 residents and 4082 housing units [24,25]. The Qualla Boundary primarily has mixed hardwood forests with some softwoods and is influenced by periodic fires [24,26].

### 2.2. Sampling

Samples for this study were collected from 25 coyotes shot by wildlife control personnel on the Qualla Boundary over the period of November 2016 to June 2018 (Table 1). The animals were dissected, and the stomachs were removed, their ends tied with string, and frozen upon collection.

### 2.3. Morphological Identification

Frozen stomachs were thawed and dissected in the autopsy laboratory of the Department of Fish and Wildlife Conservation at Virginia Tech University in Spring 2019. Items in stomach contents that could be identified morphologically by expert recognition or the use of a dichotomous key [6] were recorded. Samples of animal origin that could not be identified visually were taken for molecular genetic analysis.

### 2.4. DNA Barcoding

DNA was extracted from animal-derived stomach content samples using the DNeasy Blood and Tissue Kit (Qiagen, Germantown, MD, USA) using the manufacturer’s protocol. The mitochondrial cytochrome oxidase I gene was amplified using the framework outlined for the routine barcoding of mammalian samples by Ivanova et al. [27] with a cocktail of eight primers (LepF1_t1, LepR1_t1, VF1_t1, VR1_t1, VF1d_t1, VR1d_t1, VFli_t1, and VRli_t1). Polymerase chain reactions (PCRs) contained 6.25 μL 10% trehalose, 2 μL of distilled water, 2.5 μL of 5x PCR buffer (Promega, Madison, WI, USA), 1.25 μL of MgCl_2_ (25 mM), 0.125 μL of forward primers (0.01 mM) and 0.125 μL of reverse primers (0.01 mM), 0.25 μL of dNTPs (2.5 mM), 0.06 μL of Taq DNA polymerase (Promega), and 2 μL of DNA template, for a total reaction volume of 15 μL. The PCR was completed in a MyCycler (BioRad, Hercules, CA, USA) with an initial denaturation at 94 °C for 2 min; followed by five cycles at 94 °C for 30 s, 50 °C for 40 s, and 72 °C for 1 min; followed by 35 cycles of 94 °C for 30 s, 55 °C for 40 s, and 72 °C for 1 min; followed by a final extension at 72 °C for 10 min. The PCR products were then cleaned of unincorporated nucleotides and primers with ExoSAP-IT (Applied Biosystems, Foster City, CA, USA). Samples were then sent to the Fralin Life Sciences Institute Genome Sequencing Center (GSC, Blacksburg, VA, USA) for Sanger sequencing. Forward and reverse sequences were aligned into a contiguous sequence using Geneious Prime 2020.0.5 for Windows software (Geneious, Auckland, New Zealand). The Basic Local Alignment and Search Tool (BLAST) [28] was used to compare the resulting sequences to highly homologous sequences archived in GenBank (https://www.ncbi.nlm.nih.gov/nucleotide/, accessed 1 May 2020). Identifications were considered positive with a homology over 97% over a run of at least 200 nucleotides. 

## 3. Results

### 3.1. Morphology-Based Findings

Morphological observation indicated that all coyote stomachs contained food items, with every stomach containing some type of animal material and 19 of 25 stomachs containing some type of vegetal matter (Table 1). Vegetal-derived items included foods from the wild (leaves, seeds, and pine needles), human plantings (crab apples), and human refuse (sliced carrots, corn, sunflower seeds, scallions, tomatoes, peanuts, and squash). Similarly, morphologically identified animal-derived items included foods from the wild (worms, small and large mammals, and insects), food from human sources (eggshell), and food of uncertain wild or human sources (pork; feral pigs are present in the Qualla Boundary [29]). Notable findings included a large tooth from a predator in individual 15 and the lower jaw and teeth of a small black bear (*Ursus americanus*) in individual 25. At least seven coyotes had clearly consumed material from human trash.

### 3.2. DNA Barcoding-Based Findings

DNA from 11 animal-derived food items that could not be readily identified morphologically was successfully amplified, sequenced, and the results identified by comparison against DNA sequences of known sources archived in GenBank. Of the samples identified (Table 1), four were pigs/hogs (*Sus scrofa*), four were cattle (*Bos taurus*), one was a chicken (*Gallus gallus*), and two were coyotes (*Canis latrans*). Of the 11 samples, 2 from individual 8 were both identified as *S. scrofa*, and 2 from individual 11 were both *B. taurus*. Identifications of coyote DNA could have been derived from cells sloughed from the digestive tract of the host. Animal-derived foods included chicken and beef clearly derived from human sources, as well as pork that could have been derived from the wild or from human sources.

## 4. Discussion

### 4.1. Background to Our Study

To our knowledge, no one has ever systematically surveyed mammals on the Qualla Boundary. However, coauthor D.L. has worked in the adjacent Great Smoky Mountains National Park since 1963 and has a total of over 32,000 trap nights of effort there. Small mammal trapping by coauthor D.L. and others in the National Park between 2002 and 2010 resulted in considerably lower numbers of small mammals than experienced in many years of trapping there. For example, in 2002, a total of 660 trap nights yielded 11 small mammals along the Roaring Fork Motor Nature Trail (Sevier County, Tennessee). Trapping in August 2003 in the vicinity of the Tremont Institute (Sevier County, Tennessee) yielded just four small mammals in 100 trap nights; in 2004, 85 trap nights yielded one small mammal); and in 2005, 80 trap nights yielded no small mammals. During the summer of 2004, trapping was conducted near the junction of Rhododendron Creek Trail and Grapeyard Ridge Trail in the Greenbrier area of the National Park (Sevier County, Tennessee). This effort of 180 trap nights resulted in the capture of just one short-tailed shrew (*Blarina brevicauda*). A small mammal study during 1999–2003 yielded a capture rate of 0.046 (36 small mammals in 784 trap nights), and one conducted in 2010 yielded a capture rate of just 0.007 small mammals (21 specimens in 3206 trap nights) [21]. Those authors noted that coyotes were first observed in the National Park in 1982; although, they may have occurred in the park as early as the 1940s [5]. It is an opportunistic predator that consumes a wide variety of prey items according to seasonal availability, with small mammals consistently constituting a portion of its diet [30]. Kumar et al. [21] noted that predation by *C. latrans* may have contributed to a small mammal decline in the National Park.

As noted above, the Cherokees began reintroducing white-tailed deer onto the Qualla Boundary in 2014, and the wildlife manager was interested in whether or not coyotes were preying upon deer.

### 4.2. Diet of Coyote on the Qualla Boundary

The data collected on coyotes on the Qualla Boundary in North Carolina are consistent with those of studies in other areas in suggesting that coyotes consume a diverse, omnivorous diet including food items obtained from both natural ecosystems and anthropogenic sources [11,12,13,14,15,16]. Both morphological and DNA barcoding results found that human refuse played an important role in providing protein to coyote diets and did not indicate a significant dependence on native mammals. We could not conclusively determine whether pork was from human food waste or from predation upon feral pigs (*Sus scrofa domesticus*) or wild hogs (*Sus scrofa*). Feral pigs and wild hogs are both present in North Carolina [29], and Jensen et al. [16] observed greater consumption of feral pigs by coyotes in the spring and winter when all the stomachs that contained *Sus scrofa* items were collected. However, most samples of animal origin that we identified to the species level were domesticated animals used for human consumption. Beyond the genetically identified meat items, the importance of human-derived food items was supported by observations of a foil wrapper, sliced carrots, corn, sunflower seeds, and peanuts, all of which would be highly unlikely to occur in the ecosystem without the influence of humans. Understanding the biomass contributions of different food sources would help provide clarity to the relative importance of different foods in coyote diets, but DNA barcoding is able to reliably report only whether a species is in a sample, rather than how much of each species is represented [31]. We observed evidence of the consumption of small mammals in three of the stomachs that we examined; that wild sources of animal-derived material were recovered in only a few individual coyotes suggests that native wildlife does not play a dominant role in the diet of Qualla Boundary coyotes. While the presence of coyotes may have altered ecosystem dynamics and led to small mammal declines, our results do not suggest that such declines are a direct consequence of ongoing heavy predation or reliance upon those wild animals. An assessment of whether this level of predation can explain the decline of small mammals locally would require the examination of the contents of more coyote stomachs and a demographic simulation of prey populations.

Scat-based diet studies in nearby states found that the most prevalent meat items in coyotes’ diets were white-tailed deer and small rodents [32,33,34,35,36]. While there was no identifiable evidence of white-tailed deer consumption in our study, the low percentage of successful PCR-based item identifications raises the possibility that some of the unidentified samples could be *O. virginianus*. Additional factors that may contribute to the lack of identifiable *O. virginianus* samples in this study could be due to samples being collected outside of seasons when coyotes consume the most deer [17], low numbers of deer within the Qualla Boundary [37], and evidence that deer consumption is lower in developed areas [38].

The conclusions of this study are limited by the small sample size (*n* = 25) and the low frequency of samples successfully identified through DNA barcoding (13.5%). It is also possible that the role of human refuse is overestimated in our results because the individual coyotes analyzed were all culled due to human–wildlife contact. On an individual level, the role of human-derived foods in specific coyotes’ diets may have been overestimated by recent foraging in human refuse. The role of human-derived foods may also have been overestimated on a population level because there is variation in boldness and inclusion of anthropogenic food sources among individual coyotes [39,40,41], and coyotes included in this study may represent a bolder portion of the population. As a result of these limitations, our results do not conclusively catalogue the exact proportions or full breadth of coyote diets in the Qualla Boundary but rather indicate that coyotes within the Qualla Boundary have diverse, omnivorous diets that contain human-derived foods.

Despite these limitations, the data we collected are consistent with the results of other diet studies in the American Southeast suggesting that coyotes consume a diverse, omnivorous diet including food items obtained from both natural ecosystems and anthropogenic sources [11,12,13,14,15,16]. The occurrence and proportions of various food items in coyotes’ diets change with seasonal and regional availability [14,15,16,32,33]. A study of coyote stomach contents collected in Tennessee [34] showed rodents, rabbits, deer, and persimmons (*Diospyrus virginia*). DNA barcoding of items in coyote scats collected in Virginia [35] showed 21 mammal species including cattle, sheep (*Ovis aries*), and black bears; birds, reptiles, insects, soft mast, and hard mast were also found in that diet study. The full impact of coyotes on ecosystems in the American Southeast remains unknown.

### 4.3. Ecological and Management Implications

Human developments have provoked a range of responses—including avoidance, adaptation, or exploitation—in carnivores, but the carnivores that seem to be the most suited to urban areas are dietary and ecological generalists [42,43,44,45]. Besides coyotes, generalist carnivores like caracals, pumas, bobcats, and red foxes have found successful strategies to deal with urbanization [42,43,45,46]. A commonality across these species is the opportunistic consumption of abundant resources and trophic flexibility [42,43,45,47,48]. Some urban carnivores consume more prey at lower trophic levels compared to their wildland counterparts [42,43,45,47]. The absence of traditional apex predators, such as wolves (*Canis lupus*), has also newly moved coyotes and other urban carnivores into an apex predator position [47]. The dietary and behavioral flexibility of carnivores that exploit urban areas leads to lots of variation among locations, and a variety of studies are necessary to understand the repertoire of strategies they employ. Coyotes are unique among carnivores in urban areas by not only tolerating but also actively seeking out urban areas; one study noted a higher prevalence of coyotes in increasingly urbanized areas, in contrast to patterns observed in other carnivores [48,49].

Coyote social structure and demographics have been well studied over a wide range of habitats throughout the United States and Canada [50]. Coyotes live in packs, are territorial, and have a strong social hierarchical structure in which typically only the alpha pair breeds within each pack [51,52]. Each pack occupies and defends one territory [51,53,54]. Territories are typically contiguous [52,54,55,56], with each territory maintained by an alpha pair [57,58]. Packs usually also contain beta coyotes, which are typically related to the alpha pair, and pups. The larger population also contains transient coyotes [51,53] that occupy the interstitial area between several territories [59] and generally do not produce offspring [60]. A disruption of the social structure can lead to shifts in coyote behavior. For example, the death of the alpha male and subsequent temporary abandonment of the territory by the alpha female in the Lamar Valley in Yellowstone National Park, USA, led one of the three packs to shift its space use into part of the adjacent pack’s territory and to maintain occupancy of the newly acquired area even when the alpha female returned with a new mate [61]. Neither food shortage nor prey availability was a contributing factor. However, virtually all decisions about predator management occur in the face of incomplete knowledge of the often spatially and socially structured environment and in systems subject to temporal variation [50]. Pitt et al. [62] constructed an individual-based stochastic coyote population model that incorporated social structure via pack rules, and Conner et al. [50] developed a spatially explicit, individual-based simulation model to evaluate commonly used or promoted coyote control strategies. The latter model incorporated behavioral features, such as dominance and territoriality, and a spatial component, and enhanced the social rule set to model coyote movement and territory replacement. Model results suggested that the spatial strategy of intensively removing coyotes from a reduced area is more efficient than random removal and produces better results, especially considering that it resulted in a greater reduction in the number of alpha coyotes. In addition, the reduction in coyote numbers lasted longer, which is important for reducing control costs. In the context of this study, where the Qualla Boundary has an area of only 213.9 km^2^, the removal of scores of individual coyotes over a period of years longer than our study poses impacts on pack social structure. This in turn can lead to the increased movement of individual coyotes and increased negative human–coyote interaction.

Relatively little research has been conducted to help managers and the public make informed decisions about managing urban coyote conflict [63,64,65]. The reactive management of coyotes, including culling, has been widely practiced, though its efficacy has been questioned. Trapping is an effective tool in removing problematic coyotes and re-instilling the fear of humans in most cases; however, calling and shooting by well-trained personnel are also an important management approach and sometimes the only option [66]. Behavioral aversion conditioning, or humane hazing, is increasingly advocated as an effective and compassionate alternative to wildlife management strategies, such as traps and removal [65,67]. Breck et al. [68] concluded that the reactive lethal removal of problematic coyotes in the Denver, Colorado, area was an effective means of managing conflict, and that the hazing of problematic coyotes was largely ineffective at reducing human–coyote activity overlap. In Toronto, Ontario, Canada, a coexistence policy focuses on public education as a way to shift perceptions, increase the knowledge of coyotes, and modify human behavior (especially regarding waste disposal and property maintenance) to dissuade coyotes from habituating to human-provided food sources [69]. In our context, the resolution of human–wildlife conflict on the Qualla Boundary could include adaptations by the human population to coexist with these invasive carnivores. Notably, coyote foraging on human refuse could be managed by the adoption of scavenger-resistant garbage containers. To investigate the potential for human–coyote coexistence in urban environments, Elliot et al. [70] surveyed residents of Chicago and Los Angeles regarding residents’ opinions, fears, knowledge, personal experiences with urban coyotes, and behaviors affecting them; the results showed great variation in attitudes towards coyotes, with animal lovers being as much of a problem as those with a paralyzing fear of wildlife. Hence, finding acceptable solutions may pose a significant challenge to urban wildlife managers and reconciliation ecologists. Tailoring messages to audiences based on prior experience with coyote problems may improve the efficacy of communication campaigns designed to reduce problem interactions with coyotes [71].

## 5. Conclusions

As coyotes colonize eastern North America, they adapt to exploit a wide range of natural and anthropogenic food resources. Morphological and molecular examinations of stomach contents from coyotes culled within the Qualla Boundary of western North Carolina showed a wide variety of plant and animal items of both natural and anthropogenic origin. Our observation of native mammal remains in 3 of 25 stomachs suggests that coyotes could impact native mammal populations, which have been subject to recent decline. Our observation of diet items derived from human food waste in 8 of 25 stomachs indicates that coyotes forage on trash, suggesting change is needed in human behavior to adopt predator-resistant trash containers and thereby manage human–wildlife conflict.

## Figures and Tables

**Table 1:** Morphological and genetic identification of items observed in coyote stomachs collected on the Qualla Boundary, NC, USA. Each row is a single coyote included in the study. Morphologically identified plant and animal materials are listed first. Unknown materials recovered during dissections underwent DNA barcoding, and samples that returned an identification are listed under genetic ID. Comments generally categorize contents into native animal sources or anthropogenic food sources when these categorizations were immediately clear. Question marks in column 3 indicate uncertain identifications.

No.	Date	Morphological Identification		Genetic ID	Comment
	Collected	Plant Material	Animal Material	Animal Material	
1	5 December 2016	-	Brown mass		
2	1 February 2017	-	Worms, parasites	*Canis latrans*	Likely host material
3	31 January 2017	-	Bones, teeth, hair, skin		
4	5 January 2017	-	Mouse skull		Native mammal
5	11 January 2017	-	Unknown material		
6	7 November 2016	-	Pale material, hair	*Canis latrans*	Likely host material
7	15 May 2018	Two types	Unknown material		
8	31 January 2017	Sliced carrot, crab apples, leaf	Unknown material	*Sus scrofa*	Foraged human trash
9	28 June 2018	Grass	Unknown material	*Bos taurus*	Foraged human trash
10	10 June 2018	Pine needles	Unknown material, bone, fat		
11	15 May 2018	Vegetation	Unknown material	*Bos taurus*	Foraged human trash
12	9 January 2018	Leaf	Small mammal		
13	31 January 2017	Leaf, pine needles	Pork?		
14	11 September 2017	Seeds, crab apple	Dragonfly wings, unknown material		
15	26 January 2017	Seeds, grass	Big tooth from predator, unknown material, caterpillars		Native mammal
16	15 November 2016	Seeds, pine needles	Unknown material, fly		
17	31 January 2017	Corn, plant materials, sunflower seeds, leaf	Unknown material	*Gallus gallus*	Foraged human trash
18	14 November 2016	Corn, grass, leaf	Small mammal, mouse skull		Foraged human trash
19	28 June 2018	Pine needles	Unknown material	*Bos taurus*	Foraged human trash
20	24 January 2018	Grass, corn, scallion, foil wrapper, tomato skin	Unknown animal material—bone, membrane, white tissue, eggshell	*Sus scrofa*	Foraged human trash
21	16 April 2018	Peanuts, leaf	Unknown animal material, incl. bone; caterpillar		Foraged human trash
22	31 January 2017	Seeds, pine needles	Pork? bone		
23	15 May 2018	Leaf	Hair, intestine		Scavenged a gut pile
24	22 November 2016	Grass, leaf	Black hair from bear?		
25	24 January 2017	Corn, squash	Pork, with hair? Rabbit? Lower jaw and teeth of a small black bear	*Sus scrofa*	Native mammal

## Data Availability

The original contributions presented in the study are included in the article, further inquiries can be directed to the corresponding author.
